# Clinical Simulation in the Training of Obstetrics and Gynecology Resident from the Perspective of Medical Residency Programs

**DOI:** 10.1055/s-0043-1770127

**Published:** 2023-06-20

**Authors:** Simone Pereira Vidotti, Nildo Alves Batista

**Affiliations:** 1 Universidade Federal de São Paulo, São Paulo, SP, Brazil

**Keywords:** simulation training, internship and residency, obstetrics and gynecology department hospital, health human resource training, patient safety, treinamento por simulação, internato e residência, unidade hospitalar de ginecologia e obstetrícia, capacitação de recursos humanos em saúde, segurança do paciente

## Abstract

**Objective** This study analyzes the role of clinical simulation in internal medical residency programs (IMRP) in Obstetrics and Gynecology (OB/GYN), attributed by the supervisors, in the training of residents in the city of São Paulo (SP).

**Methods** Cross-sectional descriptive, qualitative, and exploratory approach. Semi-structured interviews were performed with ten supervisors of Medical Residency programs in Obstetrics and Gynecology. Interviews were analyzed by means of content analysis under the thematic modality, starting with the core *the role of clinical simulation in Obstetrics and Gynecology Medical Residency Programs.*

**Results** Supervisors view Clinical simulation as: a complementary tool for the teaching and learning process, a possibility of a safe teaching and learning environment, an opportunity to learn from mistakes, a support for professional practice committed to patient safety, a learning scenario for teamwork, a scenario for reflection on the work process in Obstetrics and Gynecology, a scenario for evaluative processes in the medical residency. Still according to supervisors, Clinical Simulation favors decision-making and encourages the resident participation in activities.

**Conclusion** Supervisors recognize Clinical Simulation as a powerful pedagogical tool in the learning process of resident doctors in Obstetrics and Gynecology Residency Programs.

## Resumo

**Objetivo** O presente estudo analisa o papel da Simulação Clínica em programas de Residência Médica (PRM) de Obstetrícia e Ginecologia, atribuído pelos supervisores, na formação do residente no município de São Paulo (SP).

**Métodos** Abordagem qualitativa, transversal, de natureza exploratória e descritiva. Foram realizadas entrevistas semiestruturadas com dez supervisores de programas de Residência Médica de Obstetrícia e Ginecologia. Para análise das entrevistas foi realizada análise de conteúdo na modalidade temática partindo do seguinte núcleo: o papel da simulação clínica nos Programas de Residência Médica de Obstetrícia e Ginecologia.

**Resultados** A Simulação Clínica, na visão dos supervisores, emerge como: ferramenta complementar para o processo de ensino e aprendizagem; possibilidade de um ambiente de ensino e aprendizagem seguro; possibilidade de aprendizagem a partir do erro; suporte para prática profissional comprometida com a segurança do paciente; cenário de aprendizagem para o trabalho de equipe; cenário de reflexão sobre o processo de trabalho em Obstetrícia e Ginecologia; favorecimento na tomada de decisão; cenários de processos avaliativos na residência; e, por fim, estímulo à participação dos residentes nas atividades.

**Conclusão** Os supervisores reconhecem a Simulação Clínica como uma ferramenta pedagógica potente no aprendizado dos médicos residentes em Obstetrícia e Ginecologia.

## Introduction

Historically, traditional methodology based on Cartesian thought has guided the education of health professionals, marked by a fragmented and reductionist approach. The search for technical efficiency and for specialized knowledge has led to the emergence of several changes within the educational institutions as well as on the educational propositions. Such changes have equally produced effects in the teaching and learning dynamic, in which the lecturer performs as a content transmitter while the student just plays the role of a spectator. A system that remained unaltered for the past 100 years, notwithstanding the important changes in healthcare. 1

Lately, there has been a change from the traditional Halstedian training model – “see one, do one, teach one” – to a more contemporary model of Based in Competence Medical Education – BCME. A Medical Education founded on competencies becomes popular all over the world as a new approach in education and evaluation of the novice physician. 2 3 4

The Entrustable Professional Activity – EPA – a concept brought about by Ten Cate and Scheele, 2007–emerges within that context to fill in the gap between competence-guided education and the clinical praxis. 5 In clinical practice the competences are intertwined in a complex way so that they are less explicit and measurable. A reliable professional activity is one that may be entrusted to a person once that person has achieved the necessary competence. The EPAs represent the professional's daily activity, which means they are observable, measurable entities that can be the focus of evaluation. 6

Therefore, thinking that teaching-and-learning process within a perspective of construction of knowledge – in which resident and professor take effective participation – implies vertically substituting both the memorizing-of-information process and the fragmented transfer of knowledge by a praxis that gathers knowledge through an interdisciplinary posture. In that regard, one values the adoption of methods that encourage students to effectively participate throughout the process. The simulation method is among those known as active methodologies. 7

Medical simulation may be an ancient art. However, it is a young science that has just held a position at higher education institutions. 8 Simulation uses technology and has tools like simulators, and yet these last ones do not encompass the meaning of simulation despite of being part of it. 9

Simulation also favors the development of competencies related to clinical procedure pertaining to the professional praxis. It also goes beyond the technical and technological aspects to reach the development of analysis, synthesis, and the decision-making process. In the United States, Canada, and Europe, several higher education institutions have simulation centers where that methodology is explored and widespread.

In Brazil, it is possible to notice a greater adhesion to simulation from private and public institutions, as well as an increasing tendency to build simulation centers. However, the high costs demanded to build facilities, to acquire simulators, and to hire skilled personnel seem to hinder that expansion. Notwithstanding those factors, simulation has become popular in the medical field as a complementary means to the traditional training in patients, by improving the abilities while favoring doing “the real thing” in a safe learning environment.

While pondering the national scenario regarding the use of Clinical Simulation within the medical postgraduation courses, a worry emerged concerning the way that tool is employed throughout the Medical Residency Programs, specially those of Obstetrics and Gynecology. The primary assumption was that Clinical Simulation is comprehended by supervisors of the Medical Residency Programs of Obstetrics and Gynecology as an effective pedagogical tool in the residents learning process, though not very used. Therefore, the purpose of this study was to analyze the role given by the program supervisors to the Clinical Simulation applied to the training of residents in Obstetrics and Gynecology in the city of São Paulo.

## Methods

A cross-sectional descriptive, qualitative, and exploratory study was conducted. The research took place in the city of São Paulo by interviewing 10 program supervisors among 18 who were present at data collection time. The physicians interviewed supervise a total of 358 residents, 72% of the total number of trainees in Gynecology and Obstetrics in the city of São Paulo. As of the seventh interview, a saturation point of data collection was noticed once information reoccurred. However, it was decided to continue interviewing up to the tenth interview aiming to gather a diversity of institutional features.

First section of data collection used a questionnaire composed of closed questions to characterize the survey participants. The second section consisted of an interview intended to apprehend the role supervisors ascribed to clinical simulation.

Data analysis of the literal transcription of the semi-structured interviews was performed and the results were analyzed by means of a three-stage content analysis namely pre-analysis, exploring the material, and treatment of results. Pre-analysis involved a fluctuating reading of all transcripted material obtained from the interviews, which allowed a better comprehension of the context as well as assimilation of impressions and trends that were found. 10 A session of repeated reading of material was followed by the identification of Context Units (CU), that was guided by the core theme *The role of simulation in OG Residency.* CU are understood as broader and more contextualized parts of all that was said related to that theme, and that was considered essential to the necessary analysis and interpretation of texts to be deciphered. 11 Based on the CUs one could get to the Register Units (RU) as “the smaller part of content whose occurrence is registered according to the categories found. 11 ”

A categorization process followed the defining of UC and UR. Categorization process is understood as “a classification operation of constituent elements of a set by differentiation, followed by an analogy-based regrouping according to defined criteria. 11 ” To get to the categories and subcategories the semantic process was applied by grouping the RUs interpretations. Both categories and subcategories came forth from what was said by the interviewees. 11

## Results and Discussion

OG Residency Programs of diverse natures were included, such as those from universities, and from nonprofit hospitals owned by federal, municipal or state public administration, as well as from philanthropic hospitals. Each participant received an interviewee's code that ranged between 1 and 10 to assure anonymity. Among the institutions, six are public and four are philanthropic. As for the number of vacant posts accredited at the Medical Residency National Committee (MRNC), the average was 12 vacant posts per year (six at minimum and 20 at maximum). All participant institutions either hold their own medical internship program or provide a training field to another institution's internship. Characterization is presented on Table 1 .

**Table 1:** Characterization of the Institutions which participated in the research

Characteristics	n	%
Administrative Category		
Public	6	60
- Federal	1	10
- State	4	40
- Municipal	1	10
Philanthropic	4	40
Academic Organization		
University	5	50
Nonprofit Hospital	5	50
Number of accredited OG MR vacant posts Vacant posts / year (Mean)	12	
Medical Internship		
Yes	10	100

As for the supervisors' profiles, most of them were male doctors, aged between 40 and 50, with an academic title, as one can see on Table 2 .

**Table 2:** Characterization of Supervisors who participated in the research

Characteristics	n	%
Age (years)		
< 39	1	10
40–49	6	60
50–59	2	20
> 60	1	10
Gender		
Male	7	70
Female	3	30
Time undertaking their duties (years)		
< 1 year	2	20
1 a 5 years	4	40
5 a 10 years	3	30
> 10 years	1	10
Academic title		
Specialist	4	40
Master	1	10
Doctor	5	50

As initial findings all supervisors considered that Clinical Simulation plays a relevant role in Obstetrics and Gynecology Medical Residency Programs, according to what is said in the following transcript:

*Personally, I consider Realistic Simulation very important...quite inexorable, a matter of time to evolve to that point [E6* ]. As for the acquisition of abilities, some research indicate that simulation could be superior to traditional medical. 12 Those professionals who work as OG educators must study simulation and certainly embody it in their students and residents educational processes. 8 In the United States the use of simulation is among the criteria set to accredit Medical Residency Programs, which corroborates the importance of extensively using it to improve performance of specialists during their technical procedures. 13 Analysis of interviews identified 58 context unities and 78 register unities. Among the register unities, 9 categories and 11 subcategories emerged, according to Chart 1 .

Simulation appears as a **teaching and learning process complementary tool** in OG Residency, able to assist in the resident professional development. In the last decades, OG international and national societies have encouraged the use of Simulation as a complementary tool in the teaching and learning process. In 2007 the American College of Obstetricians and Gynecologists (ACOG) acknowledged simulation as a valuable educational element in undergraduate and graduate studies. Simulation-based methods offer medical students the opportunity to obtain key qualities at the working place, such as confidence, knowledge, skills, and the appropriate behavior able to offer a high-quality service to the patient within a safe learning environment. 14 15

Among the highlighted options, supervisors emphasized *that simulation may homogenize teaching and learning opportunities.* Thereby, the use of simulation seems to be significant, specially nowadays when health services make changes in health care while reduce length of hospital stay, which limits bedside learning opportunities. Such circumstance entails curtailment of occasions when residents could be in touch with risky situations and procedures. 16

*The possibility of training rare procedures* was also emphasized by supervisors. Simulation may protect against unnecessary exposure to a variety of situations, which represents an increasing need due to limited clinical training opportunities. 11

**Chart 1:** Core theme categories and subcategories: the role of simulation in obstetrics and gynecology medical residency programs

Category	Subcategory
Teaching and learning process complementary tool	Teaching and learning opportunities homogeneity possibility
	Less common procedures training possibility
	Unlimited repetition of procedures
	Residents self-confidence training
Safe teaching and learning environment possibility	
Learning from error possibility	Improving performance by repeating the undergone experience
Support for professional practice committed to patient safety	
Teamwork learning scenario	
Reflection scenario on the Obstetrics and Gynecology working process	Discussion about multidisciplinary care/assistance protocols
	Preparation for safer professional practices aiming at reducing judicial risk
Decision-making support	
Residency appraisal process scenario	Recruitment process scenario for admission at medical residency
	Possibility to appraise multiple skills expected from health professionals during Medical Residency Programs
	Possibility for Interactive feedback
Encouraging residents participation in the Medical Residency Programs activities	Practical performance improvement

Supervisors also emphasized *the possibility of unlimited repetitions of procedures* . Simulations may also allow deliberated practice, which could be defined as the engagement of students in repeating the abilities thoroughly, focusing on progressive exercises and informative feedback. 17

Deliberated practice is essential in cases whose procedures are so rarely performed that few professionals could actually master the necessary abilities without having practice and feedback at a non-clinical environment. Such rare procedures have usually been associated with high-risk situations, which lead to medical errors. Deliberated practice performs a main role in preparing professionals for critical events, 18 besides being regarded as a most powerful indicator of the specialist's performance when compared with experience and academic aptitude. 19

Supervisors also emphasized *the residents' self-confidence training* , as it allows greater confidence in their abilities. Humes et al report that resident doctors felt more confident about their abilities after performing a vaginal hysterectomy training in a uterus model by using a sponge and a PVC pipe. 20

According to the interviewees, the possibility to have a safe teaching and learning environment offers calm conditions to the residents as they do not feel pushed to be perfect at performing or even not to make errors. The possibility to ensure a protected environment in which residents may perform tasks, detect errors, and correct them without producing adverse consequences, and where instructors may find the opportunity to connect better with their apprentices and techniques, is one of the elements which contributes to effectiveness in simulation. 21

In this context, the possibility of learning form errors minimizes the trouble of dealing with that matter in real practice before the patients. It helps *improving performance by experience repetition* until attaining the goal. To Maslovitz, simulated training allows thus identifying and correcting common clinical errors made during emergencies. 22

Supervisors understand that clinical simulation provides support to a professional practice that is committed to patient safeness. Evidence shows that obstetricians have improved their technical and communication abilities by practicing. In that sense, programs, which concern patient safety, must incorporate Obstetrics and Gynecology simulation. 23

Sustained and increasing focus on medical error reduction and on patient safeness, as well as the need to offer a safe, ethical, and student-centered training lead to a model, which incorporates Simulation-based Education. 18

The role of simulation has also been described as a scenario for teamwork in which it is emphasized its application in multidisciplinary training as well as in Permanent Education.

Training patterns for quick response in obstetrics emergencies are useful to improve team performance and bring better results to patients. 24 A systematic review on simulation-based training evaluation determined that teamwork became more efficient not just due to advancement of scientific knowledge, but also due to improvement in both communication skills and obstetrics emergency management. 25

Simulation-based education proved itself as a Scenario for reflection about OG work process. It is important to highlight that failure to communicate in teamwork contributes to most obstetrics sentinel events. Labor pains and labor itself are critical moments when emergencies occur.

The American College of Obstetricians and Gynecologists – ACOG (2014) states that the care provided in emergency cases is enhanced by protocols that have standardized interventions and that promote on-the-job training. Team may learn and practice the necessary interventions while improve efficiency and reduce errors. 26

Within this context, simulation may be used to *discuss multidisciplinary protocols of assistance* . As an example, a pilot study using simulation identified ∼20 flaws in the safe application of a new intraoperative radiotherapy procedure before testing it out in patients. Such procedure included radiation safety, teamwork, team communication, and problems with both equipment and supply. 27 Thus, simulation was a scenario for the creation and discussion of a patient safeness protocol whenever innovation is brought to clinical environment.

Due to the increasing of lawsuits against medical practitioner's performance, supervisors stated that *preparation for safer professional practice reduces the risk of taking practitioners to court.*

According to a report by the General Council of Medicine from São Paulo (2006), professional obstetricians and gynecologists are sixth in the ranking of lawsuits. The main for these concerns the procedures related to labor assistance. Patients are usually awake when unpredictable emergencies that risk their lives occur, which makes teaching more difficult during these moments. Even experienced professionals could be surprised by both unexpected situations and rare complications that may happen during labor assistance. On account of that, medical schools and Medical Residency Programs are encouraged to develop strategies so as to avoid exposing patients to teaching under such conditions when simulation stands out as a training opportunity for students and residents. 8

Simulation highlights and enhances the role of favoring the decision-making process so as to provide the increment of professional attitudes.

A study on simulation being applied to evaluate teamwork training in decision-making process via simulation demonstrated a time reduction of 33 to 21 minutes from the indication of cesarean section to the moment of surgical incision. 28

Another role Clinical Simulation performs is that of becoming a scenario for evaluative processes in residency, by expanding the items to be evaluated within the competencies expected from professionals.

During the interviews, supervisors mentioned 3 evaluation strategies using simulation in Residency. A *scenario for the selective process enrollment* appears as a possibility. Clinical simulations allows a better evaluation of candidates as it enables a better observation of their technical abilities, in addition to their professionalism, communication, and critical thought. 29

The second strategy mentioned by the supervisors refers to the *possibility of evaluating multiple competencies expected from health professionals during Medical Residency Programs* . Simulation was thus mentioned in summative assessment as the internship completion and as the conclusion of a stage in the residency program. Although knowing about the use of simulation in evaluation, just a supervisor mentioned the OSCE model of evaluation as a preparation for the specialist diploma in Obstetrics and Gynecology. The third strategy referred by the supervisors was the *possibility of interactive feedback* , which provides an immediate and constructive response to the resident. To students, feedback represents a moment of effective learning. 30 In research done with simulation educators, Rall, Manser and Howard (2000) emphasized that debriefing is the most important part of training via simulation. One of interviewees called it “the heart and soul” of simulation-based training. 31

In conclusion, there is unanimity among supervisors as to acknowledge that simulation represents an encouragement to resident participation in Medical Residency Programs activities. They highlight resident improvement in performance while doing their practical activities. Some studies have assessed the efficacy of simulated training in student confidence, examination skills and in communication. In 2015, Smith and collaborators published a systematic review with a data meta-analysis in which a comparison between teaching pelvic examination through simulation and through traditional methods was made. The authors concluded there is an improvement in the student competence concerning pelvic examination performance, as well as in their communication abilities when the simulation method is used. 32

## Conclusion

Based on the data found in this study, OG Clinical Simulation in Residency:

- Complements the teaching and learning process, allows homogeneity of opportunities, enables less-common procedures training, a deliberate and sustainable practice as well as the resident self-confidence training.
- Provides a safe teaching and learning environment.
- Encourages trial-and-error learning which enables improvement in performance by repeating the experience.
- Favors professional practice committed to patient safeness.
- Enhances teamwork as it favors its knowledge and the development of communication abilities. Furthermore, enhances emergency managing performance.
- Encourages reflection about work process, which brings the opportunity for discussion on multidisciplinary assistance protocols, prepares for safer professional practice and reduces the risk of law suits.
- Favors the decision making process, especially in emergency situations.
- Increases evaluative processes in residency, which allows the analysis of the multiple competences expected to be found in health professionals during the Obstetrics and Gynecology Medical Residency Programs.
- Favors interactive feedback and the resulting improvement of the resident, as well as of the professor and preceptor.
- Encourages participation of residents in the activities of the Obstetrics and Gynecology Medical Residency Programs, resulting in enhancement of practical performance. Clinical Simulation is thus acknowledged as a powerful tool to be used in the residents teaching and learning process.
